# Modeling the consequences of the demise and potential recovery of a keystone-species: wild rabbits and avian scavengers in Mediterranean landscapes

**DOI:** 10.1038/srep17033

**Published:** 2015-11-23

**Authors:** Ainara Cortés-Avizanda, Maria Àngels Colomer, Antoni Margalida, Olga Ceballos, José Antonio Donázar

**Affiliations:** 1 Department of Conservation Biology, Estación Biológica de Doñana (CSIC), Americo Vespucio s/n, E-41092 Sevilla, Spain; 2 Infraestruturas de Portugal Biodiversity Chair, CIBIO-InBio. Campus Agrário de Vairão, Rua Padre Armando Quintas 7, 4485-661 Vairão, Portugal; 3 Department of Mathematics, University of Lleida, Faculty of Life Sciences and Engineering, Av. Alcalde Rovira Roure 191, 25198 Lleida, Spain; 4 Department of Animal Production (Division of Wildlife), Faculty of Life Sciences and Engineering, University of Lleida, Av. Alcalde Rovira Roure 191, 25198 Lleida, Spain; 5 Division of Conservation Biology. Institute of Ecology and Evolution. University of Bern. Baltzerstrasse 6, 3012 Bern, Switzerland

## Abstract

Restoration of demised keystone-species populations is an overriding concern in conservation biology. However, since no population is independent of its environment, progress is needed in predicting the efficacy of restoration in unstable ecological contexts. Here, by means of Population Dynamics P-system Models (PDP), we studied long-term changes in the population size of Egyptian vultures (*Neophron percnopterus*) inhabiting a Natural Park, northern Spain, to changes in the numbers of wild rabbits (*Oryctolagus cuniculus*), a keystone-species of Mediterranean ecosystems that have suffered >90% population decline after a hemorrhagic disease outbreak. Low availability of rabbit carcasses leads Egyptian vultures to extend their foraging activities to unprotected areas with higher non-natural mortality whereas growing numbers of griffon vultures (*Gyps fulvus*), a dominant competitor, progressively monopolize trophic resources resulting in a focal population decrease. Modeling shows that, even if keystone-species populations recover in core protected areas, the return to the original studied population size may be unfeasible, due to both the high non-natural mortality rates in humanized areas and long-term changes in the scavenger guild structure. Policy decisions aimed to restore keystone-species should rely on holistic approaches integrating the effects of spatial heterogeneity on both producer and consumer populations as well as within-guild processes.

The keystone species concept in ecology has a long history^1^,^2^, acknowledging the disproportionate impact that the abundances of certain populations of definite organisms may have on ecosystems. Some aspects of the application of this concept to the practice of conservation have been greatly discussed, in particular, how to narrow the definition of keystone species to avoid losing its value^1^,^2^. It is beyond doubt, however, that the rarefaction or disappearance of some taxa at any trophic level, from large predators or detritivores to pollinators, encompasses profound implications for population viability of other species and overall for the structure and functioning of food webs, communities and ecosystems (see^3^,^4^). The restoration of the populations of these species, or closely related taxa that could fulfill similar functions, is therefore an emerging research field both in ecology and conservation sciences^5^,^6^ and is considered a priority for policy managers (see^7^ and references therein).

The European wild rabbit (*Oryctolagus cuniculus*) is considered a keystone species in the Mediterranean within food webs (see review in^8^). It is the main prey for at least 29 predators (17 raptors and 9 carnivores^9^), some of which are endemic rabbit-specialists in the Western Mediterranean region (e.g. the Spanish Imperial Eagle (*Aquila adalberti*) and the Iberian lynx (*Lynx pardinus*)^10^). The sudden appearance of Rabbit Hemorrhagic Disease (RHD) during the 1990s caused a sharp decline in their populations, with rabbits disappearing from much of their former areas of distribution^11^,^12^. Consequently, numeric and functional responses of consumers were triggered. Both rabbit-specialists and mesopredator species shifted their distribution areas and altered their breeding and population dynamics, which are also shaped by an increase in intra- and interspecific competition for this scarcer trophic resource^13^,^14^,^15^,^16^,^17^. On a shorter time-scale, the wild rabbit rarefaction in wilder and protected Mediterranean ecosystems forced rabbit-consumers to forage in open humanized areas often associated with agricultural practices, where the risk of non-natural mortality is higher^18^. Additionally, the vanishing of wild rabbits, considered an important game species, led to the increase in direct and indirect persecution of predators, often through illegal methods such as poisoning^19^,^20^.

The magnitude of the problem is so serious that to counteract the negative trends in the wild rabbit populations, and consequently the declining endangered consumer species, costly conservation programs in many Mediterranean regions, often at a considerable cost, have been carried out. For example, in Spain, the European LIFE–Nature projects devoted to recovering rabbit–specialist predators (which take specific actions focused on increasing populations of lagomorphs) have invested more than 94 million Euros in these efforts^21^,^22^,^23^. Meanwhile, important quantities of public funds are also devoted to reduce direct and indirect human–induced mortality of predators and scavengers^24^. However, from an ecological point of view, these initiatives have neglected the possibility that the return to original scenarios could be detrimental, especially in Europe. Here, more intensive agriculture, resulting in major landscape transformations, forces large body-sized vertebrates to inhabit increasingly hostile environments^25^. In addition, these changes may distort the interspecific relationships within food webs^26^. In this context, it seems desirable to examine whether the recovery of wild rabbit populations would lead to increases in consumer population, similar to those seen previous to the RHD outbreak.

Here we use an empirical-bioinspired modeling approach to examine this issue using as a case study a binomial-system: the wild rabbit (*Oryctolagus cuniculus*) and the threatened Egyptian vulture (*Neophron percnopterus*). We took advantage of long-term and high-quality data collected in a Natural Park in northern Spain. There, the demise of the lagomorph population due to RHD led breeding Egyptian vultures to modify their foraging habits and look for alternative food sources (mainly livestock carcasses) in humanized areas outside the limits of the protected area where the probability of non-natural mortality increased^27^. Moreover, the scavenger guild composition has greatly varied in this region over the last few decades of the study period; the Eurasian griffon vultures (*Gyps fulvus*) are progressively outnumbering the short-bodied and less-competitive scavengers^28^,^29^. In this context, wild rabbit carcasses, previously exploited almost exclusively by Egyptian vultures, are increasingly consumed by griffons (see^30^, see below for details).

Here, our specific goals were to: i) determine the long-term viability of the population of the focal species; and to examine how Egyptian vulture population dynamics would evolve given changes in ii) wild rabbit abundance; iii) non-natural mortality in humanized areas; and iv) long-term population-trends of direct competitors (griffon vultures). To examine these issues we used Population Dynamics P-system Models (PDP)^31^. These models belong to the family of models developed by^32^ and were initially created to simulate the functioning of organelles in the cell (i.e., varying their numbers and operating parallel in a synchronous and interrelated manner). There is a close parallelism between the functioning of cells and ecosystems making P-systems an increasingly useful tool in many different research lines including animal behavior, invasion processes and conservation planning^33^,^34^,^35^,^36^.

## Results

**Figure 1:**
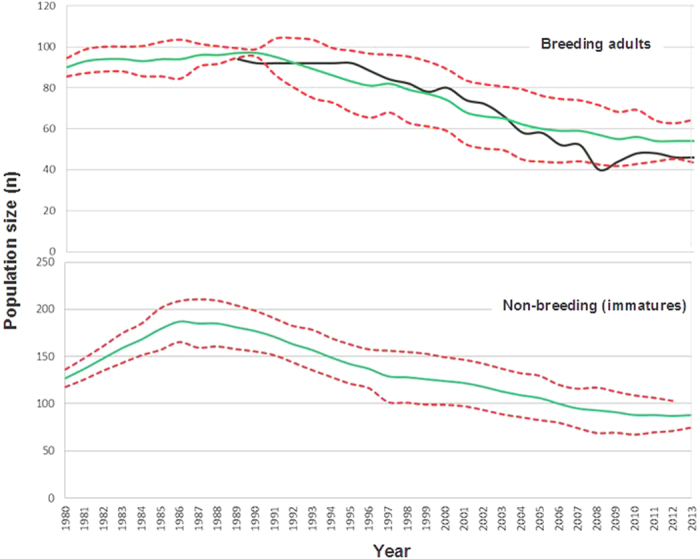
Long term changes in the numbers of breeding and immature Egyptian vultures during the monitoring period (1980–2013). Predictions derived from the basic model (green line) and empirical field data (black) are represented. Confidence Intervals are in red. No data are available for numbers of immature in the field.

**Figure 2:**
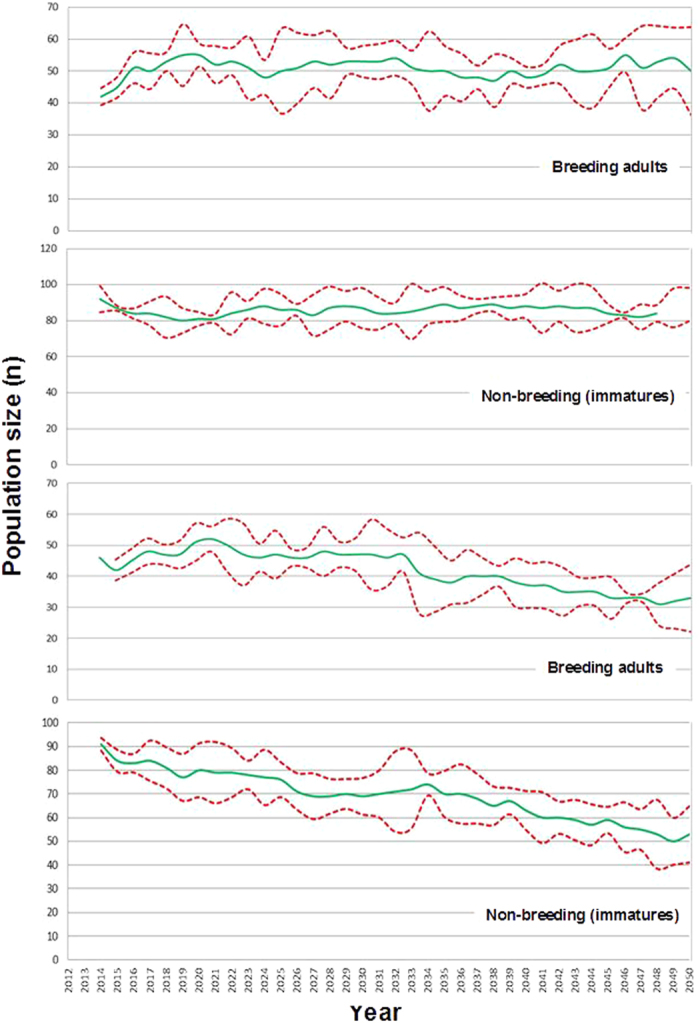
Prospective long term changes in the numbers of Egyptian vultures for a period of 35 years. Estimations are derived from parameters of the basic model. The top two graphs assume that the population of griffon vultures remains stable and the bottom two graphs that it would have doubled at the end of this period.

The projections of our model closely reproduced the observed Egyptian vulture population trend in the study area over the last three decades; in fact, the empirical data were within the 95% confidence intervals (Fig. 1). The immature fraction of the population, after a slight increase, would have decreased by almost half in the same period. Interestingly, the projections about the future trends showed that maintaining the current demographic parameters, conditions of food availability and the trophic interspecific competition with griffon vultures, the Egyptian vulture breeding population would undergo a slight increase of about 25% (from 40 to 50 adults). The number of immature birds would also increase by about 30% (Fig. 2).

**Figure 3:**
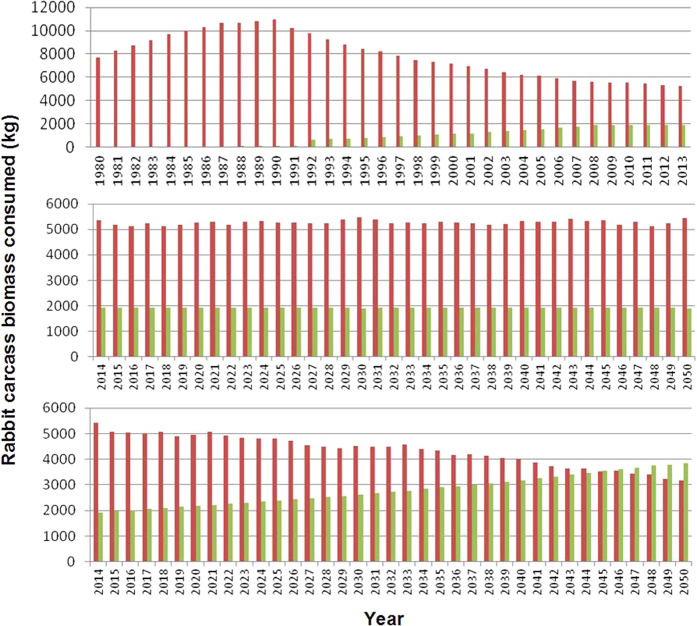
Long-term changes in rabbit biomass consumed by the Egyptian and griffon vultures. The graph above shows the estimation for the monitoring period and the middle graph the prediction until 2050. The bottom graph show the prediction until 2050 assuming that the population of griffon vultures would have doubled at the end of this period. Egyptian vulture in red; Griffon vulture in green.

Our model also estimated that the biomass of wild rabbit carcasses consumed by the Egyptian vultures since the early 1990s has continuously decreased, stabilizing in recent years. On the other hand, the number of wild rabbit carcasses consumed by griffon vultures would have gradually increased to stabilization (Fig. 3). The modeling shows that in 2013, Egyptian and griffon vultures would have consumed 73% and 27% of the available wild rabbit carcasses, respectively. Simulations of future scenarios showed that a twofold increase in the griffon vulture population in the area would occur within 35 years, and most of the griffons would consume as many rabbit carcasses as Egyptian vultures. Consequently, the models suggested a long-term population decline of Egyptian vultures (Fig. 2).

Finally, we estimated the sensitivity of the parameters (i.e., the effects of a change of 1% in their values) related to food availability, mortality by poisoning or natural mortality in a time horizon of 35 years. We found very little sensitivity to uncertainty in model projections: 0.43 more females after an increase of 1% in food availability or −1.17 and −2.04 females, after an increase of 1% in mortality due to poisoning and natural mortality, respectively.

**Figure 4:**
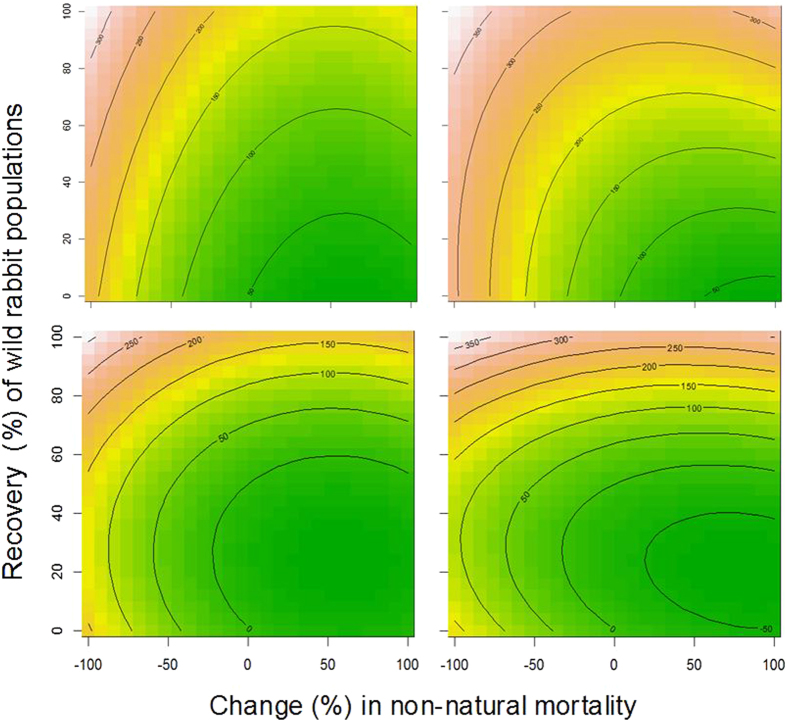
Box-Behnken projections of changes in population size of breeding and non-breeding Egyptian vultures in relation to mortality (x-axis) and the change in rabbit population (y-axis). It simulates that mortality considered in the basic model may be increased or reduced by up to 100% and that the availability of rabbit can increase up to reach 100% of the numbers existing before the RDH outbreak. The top two graphs assume that the population of griffon vultures remains stable and the bottom two that it has doubled in 2050.

Analysis using Box-Behnken procedures based on different conservation policies and management scenarios combining variations in both food availability and human-induced mortality showed that the numbers of Egyptian vultures are extremely sensitive to the latter factor (Fig. 4). Under current parameters any decrease in survival rates may lead unequivocally to population decline. Even with similar mortality rates, the models show positive population trends only if the wild rabbit populations recover by more than 40%. These projections also revealed much more positive results for non-breeding birds due to their lower sensitivity to mortality. Finally, modeling showed that if the griffon vulture populations double their population size, the recovery of the Egyptian vulture population (especially the breeding fraction) would be jeopardized even in scenarios where management actions focuses exclusively on eliminating the causes of non-natural mortality.

## Discussion

Our results show that even if the populations of wild rabbit recover, non-natural mortality of Egyptian vultures associated with the alternative exploitation of humanized landscapes would heavily condition the future viability of its population. Moreover, the modeling procedures show that the long-term changes in the composition of the scavenger guild may also play a determinant role: if the population of the dominant griffon vulture continues to increase, it would consume a significant part of the trophic resources (wild rabbit carcasses) to the detriment of the more threatened and less competitive scavenger.

Our modeling was fully parameterized from the beginning and faithfully reproduced the empirically observed long-term population trends despite the inherent simplicity of the model. This is probably a consequence of the data quality introduced into the model, which was obtained during a long-term study and allowed the true reproducibility of the process. In addition, the predictions were only very weakly sensitive to uncertainty in the parameters. However, some potentially important parameters that were not included may have additive effects that would merit consideration in future studies. This is true of the likely spatial asymmetries in the density of wild rabbits and the differential exploitation of land by Egyptian vultures living in territories of variable quality. In this regard, in some large body-sized birds of prey, home ranges varied with prey density and individual reproductive status, with habitat quality serving as a regulatory mechanism of space use (see e.g.^37^,^38^). In addition, it seems reasonable that the consumption of rabbit carcasses by vultures represents less than what our model assumes. This may be true because, apart from our focal species, there are other facultative avian scavengers and carnivores exploiting the same trophic resource^39^. Moreover, our studied population is not independent from those existing in the neighboring regions (upper Ebro Valley and Pyrenees) and may receive immigrants, which have certainly helped to slow population decline^27^ (unpublished data).

According to our findings, the priority conservation strategy is to focus on the recovery of wild rabbit populations. This goal, however, is difficult to achieve. Although rabbit populations have strongly recovered in certain areas over the last few decades, this recovery follows a very irregular spatial pattern probably due to the local environmental variability in shaping factors like vegetation cover, the presence and abundance of disease vectors, predation, and hunting pressure, whose relative contribution is still poorly understood (see^8^ and references therein). In fact, in our study area rabbit population recovery has been negligible as has been the case in other Iberian Mediterranean ecosystems^8^,^40^. Additionally, in 2012-2013 a new viral disease reached the Iberian Peninsula posing an additive risk of mortality to wild rabbit populations^41^,^42^. Thus, at least in the short to medium term, a significant increase in wild rabbit populations in this area is unlikely. In other Mediterranean regions, the scenario would be different, and vegetation succession and reforestation after land abandonment may be the rule^43^,^44^. These conditions are predicted to favor large scavengers due to the increase/expansion of wild ungulate populations^45^,^46^ but they may be less favorable for rabbit existence due to the well-known affinity of the lagomorph to open habitats^47^. Consequently, the Egyptian vulture, as well as other facultative scavengers linked to open and diverse Mediterranean landscapes, would be negatively affected by these processes contrary to large-carcass specialists like griffon vultures^46^,^48^. This scenario lends a new twist to the probability of maintenance of the populations of medium-sized avian scavengers.

Alternatively, from a demographic point of view, it is much more appropriate to focus future conservation efforts on reducing the risks of non–natural mortality in the hostile landscapes surrounding the protected areas. In our study case, the areas near the Natural Park are frequently selected by individuals because of the higher availability of carrion resources linked to the existence of both numerous farms with intensive and semi–intensive livestock operations and the development of arid lands for irrigated crops. These areas offer new habitats for small and medium–sized birds and mammals, whose carcasses could be exploited by scavengers^49^. On the other hand, in the surrounding areas the causes of mortality are mainly associated with the ingestion of poisoned baits used by local hunters and shepherds to control carnivores^27^,^50^ and with accidents involving human-made structures such as wind farms^51^,^52^,^53^. Counteracting the use of poison may be feasible in the long-term but the total eradication of this practice is unlikely without great efforts in public education^54^,^55^. The reduction of the risk of mortality at wind farms may be more attainable because these accidents follow a spatially contagious distribution^56^. To date, however, mitigating measures have rarely been implemented^57^.

Finally, our results indicate that the viability of a rabbit consumer species is not independent of the potential competitor species: griffon vultures would currently consume about 26% of the available biomass of wild rabbit carcasses. Field data corroborate this: in fact, 60% of the rabbit carcasses monitored in the study area between 2004 and 2006 were consumed by this obligate scavenger species^39^ (authors unpublished). Our models predict that if the griffon vulture population doubles in abundance this species would consume 55% of the total rabbit biomass available *vs.* 45% consumed by Egyptian vultures. Within this scenario, the probability of Egyptian vulture population decline would be higher. The growth of the griffon vulture population in the study area and throughout the Iberian Peninsula has been exponential (from 3,249 breeding pairs in 1979 to 25,541 in 2008^58^,^28^). Currently, due to lingering effects of the BSE crisis^59^ growth rates have slowed in some regions^28^ but a slow recovery was apparent in 2014. Sanitary regulations have recently changed allowing farmers to leave livestock carcasses to scavengers in the field^24^. It could be argued, however, that the Egyptian vultures also benefit from this new legislative framework as they feed on livestock remains as well, but this species depends more than other vultures on small and medium-sized vertebrates, probably due to qualitative nutrient requirements^30^,^35^. In fact, modeling outputs about population trends in relation to food shortages mediated by recent sanitary regulations (before 2011) suggested that the Egyptian vultures are far less sensitive than griffon vultures to changes in the supply of livestock carcasses^35^.

This study shows that PDP modeling procedures can be useful to determine the sensitivity of the demography of target species to changes in the behavior of individuals. Modeling strategies moving forward should aim to progress in these areas. For example, it is well–known that the degree of exploitation of a feeding place by Egyptian vultures is logically a function, though not linear, of the distance between the territory and the resource^60^,^61^). It is expected therefore, that the frequency of foraging trips outside the protected areas by the Egyptian vultures (as well as other long–ranging species) individually will depend on the spatial location of their territories. If the nests are located on the edge of natural protected areas then it is expected that the probabilities modeled here should vary (e.g. be higher). Moreover, it is also possible that there are intrinsic factors linked to each individual (sex, body size) that favor asymmetric competitive abilities and differential exploitation of space and food sources^59^,^62^. Finally, because individuals may present consistency in their behavior, asymmetries between them regarding the same behavioral traits are feasible^63^,^64^. The existence of personalities would add a new feature to the computation of survival probability of a bird performing foraging movements that involve certain variables of risks and rewards.

To conclude, although positive aspects in the recovery of threatened keystone species are widely recognized^65^, we suggest evaluating whether such recovery programs are cost–effective under changeable environmental conditions such as those supporting intensive human activity and with unstable balances in consumer populations. It has been shown that, in those systems based on a few keystone species supporting complex guilds of vertebrate consumers (whether carnivores or scavengers), the fate of the species in these assemblages may not be independent of the balance between their populations^66^,^67^,^68^,^69^,^70^,^71^. Moreover, the scenario created by the existence of protected areas within highly-humanized areas turns these into “ecological traps” where animals look for higher-quality resources but at the expense of higher mortality (see^43^ and references therein). Thus, this adds further constraints to the viability of populations of large body-sized vertebrates performing long-distance displacements.

## Methods

### Study area and monitoring of target species

**Figure 5:**
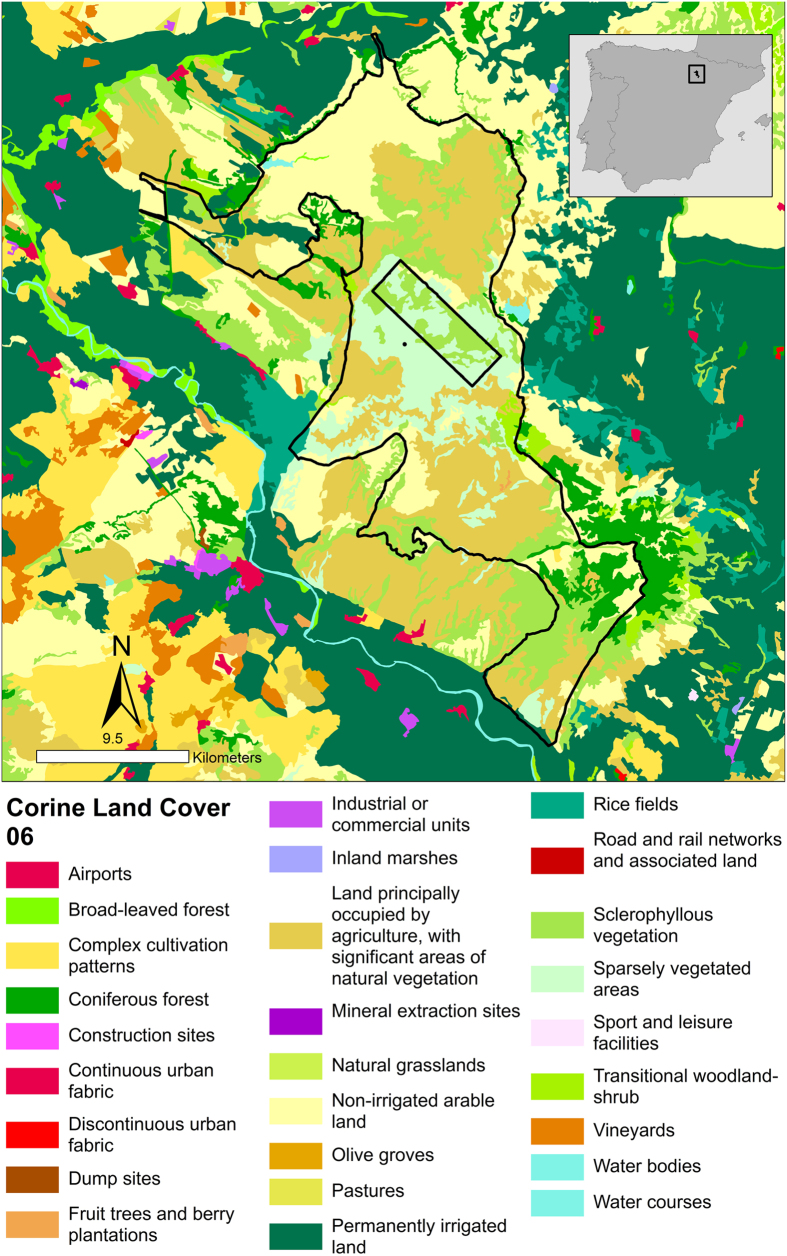
Study area: The boundaries of the Bardenas Reales Natural Park and Reserve of Biosphere (northern Spain) are shown (black line). The rectangle delimits a military area. Note that neighboring areas are densely populated (more than 150,000 persons within 30 km of the area) with numerous human localities and settlements and land uses are mainly devoted to intensive cultures. Inside the protected area there are no human settlements and dominate the non-irrigate crops, scrublands, wooded/forested patches and steppe/badlands. CLC2000-100m version 17 (12–2013).

The research was performed in the Bardenas Reales (Ebro Valley, northern Spain) (Fig. 5). This area encompasses around 50,000 ha of which around 80% have been protected since 1999 and 2000 as a Natural Park and Biosphere Reserve, respectively. This is a dry region (<300 mm/year of precipitation). It is dominated by large flat areas and small hills (280–659 m a.s.l.) with natural vegetation dominated by scrublands and small wooded patches. There are no permanent human settlements inside the Natural Park but there are large areas historically used for the traditional agricultural practices of cereal crops and grazing. In contrast, neighboring areas in the Ebro Valley are densely populated (more than 150,000 persons within 30 km of the area), and include large irrigated areas and many intensive livestock operations (see details in^27^).

Local populations of avian scavengers have been monitored annually since the early 1980s (see^27^). At that time, the density of Egyptian vultures was one of the highest known in the world with up to 50 breeding pairs (1 pair/10 km^2^). It has since declined by about 50% with only 23 breeding pairs active in 2013. During the breeding period, the Egyptian and the griffon vultures have been monitored following the well-established standard methodologies (see^27^,^72^). The annual productivity of Egyptian vultures showed variability (0.3–0.8 fledglings/occupied territory). While having been a frequent prospecting visitor, the first established breeding pair of griffon vultures was detected in 1990. Since then the population has sharply increased with 85 pairs recorded in 2013 (A. Urmeneta, unpublished data). Apart from the breeding population, there is a significant number of non-breeding birds (estimated to be of approximately similar size to the breeding fraction of the population) that forage in the same areas^29^,^48^,^73^ (authors unpublished). The annual productivity of griffon vultures has remained almost constant at around 0.4 fledglings/pair (see^27^, A. Urmeneta unpubl.).

### Model built and assumptions

In the Population Dynamics P-system Models (PDP, see Supplementary Information) we integrated information on: a) reproduction, mortality, and foraging behavior of the Egyptian vultures; b) wild rabbit carcass biomass availability, including the maximum carrying capacity of the study area; and c) carcass-sharing with direct trophic competitors (griffon vultures) (see detailed information in Supplementary Information). The model assumed that the diet of the focal species was primarily based on wild rabbits (>80%) until 1990. Subsequently, and due to the demise of the wild rabbit in the Natural Park, the birds increased their foraging in humanized areas outside of the boundaries of the protected area. As a consequence, non-natural mortality increased mainly due to the ingestion of poisoning baits and accidents with infrastructures. This led to a decrease in breeding success (associated with the loss of the mate, poor-quality of trophic resources and increased foraging effort, see Supplementary Information).

**Figure 6:**
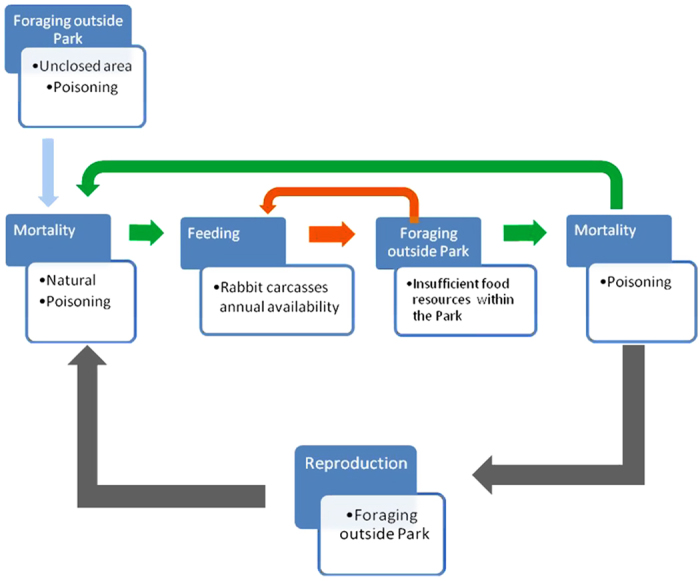
Scheme of the PDP model. The model takes into account the breeding periods (6 months) and the basic processes of reproduction, mortality, foraging behavior, as well as trophic resource availability, maximum carrying capacity and competition. The Egyptian vultures forage outside of the protected area when insufficient resources are available. Birds search for food and then return to their breeding territories. They performed more flights out of the park if the food is scarcer. The carrying capacity of the protected area is based on the biomass of wild rabbit carcasses available monthly. We consider as a unit time the day. This process run 30 times each month (N = 6) due to this is the period this migrant species spent in the study area.

The PDP model developed here is a computational modeling procedure structured according to five sequenced modules (see Fig. 6). In each module, we simulated each of the processes, with individuals evolving in parallel by means of the so-called “evolutionary rules” (see Supplementary Information for details). The first module executed the process of natural mortality and additional non-natural mortality linked to foraging movements of breeding Egyptian vultures perform into more highly humanized areas outside of the park. Birds that survive then begin the feeding process (module 2; see details in Supplementary Information). In the next module, are accounted the exits of the park for lack of food resources (module 3, how many times the individual leaves the park; see details in Supplementary Information). The foraging trips, including the potential exits of the park, are modeled daily for each month and are thus repeated 30 times. Taking the day as the unit time, modules 2 and 3 run 30 times each for the six months corresponding to the period spent by the vulture in its European breeding area. In the end, we get the number of times each animal leaves the park in search of food. This is the input for module 4, which addresses non-natural mortality due to the ingestion of poisoned baits. Once the month is completed, the model runs module 1 again and this loop is repeated six times, once for each month. At the end of the sixth month, the number of times the individual has gone in search of food on throughout the year is recorded. This parameter is the input for module 5, addressing the reproductive process. With the application of module 5 the execution of one year is complete. The loop should be repeated as many times as necessary to simulate years.

The model begins simulating an initial population in 1980 composed of 50 breeding pairs of Egyptian vultures and a similar number of non–breeding individuals, which includes birds between 1 and 5 years old. Immature birds became “breeding adults” in their eighth year of life^72^. New recruitments came from birds born during each new breeding season.

The individuals search for food daily in the protected area. Based on the data obtained from radio-tagged individuals studied in the 1980s^74^ (authors unpublished data) we consider that during this period around 20% of the foraging trips of the breeding individuals were outside the limits of protected area boundaries (i.e. in humanized areas). Before 1990, the use of poisoned baits outside the park was almost nonexistent, and therefore we consider that there is initially a cost associated with foraging behavior outside the boundaries of the Natural Park and overall mortality is taken to be 2% and 10% per year for breeding and non-breeding birds, respectively. We also considered that before 1990 the yearly productivity was 0.4 fledglings/breeding female.

We then modeled the impact of the RHD. After the outbreak in 1990, Egyptian vultures increased foraging activity outside the limits of the Natural Park. Observations conducted with tagged individuals with plastic rings (up to 50% of the total population) showed that after the demise of wild rabbits the Egyptian vultures foraged in areas up to 30 km away from their nests (unpublished authors). We assume that this implies an associated cost that leads to a decline in breeding success by more than half (up to 0.2 chicks/breeding female see^27^). In addition, foraging outside the Natural Park had other costs associated with higher mortality risk due to the occasional use of poisoned baits by shepherds, mainly in nearby hunting areas after the arrival of the RHD^27^. Consequently, the mortality rates of adult breeding birds in this population were relatively high (17% annual)^72^. This risky scenario has remained unchanged as rabbit populations have not recovered and non–natural mortality remains important outside the limits of the protected areas.

To model the role of interspecific trophic competition we estimated the number of prospecting griffon vultures in the Natural Park on the basis of: a) the density of prospecting birds estimated by^48^ and b) the regional long–term population trends of the species in the region, which has shown invariable increments during the last three decades^59^ (see Figure S2 and also Supplementary Information). Before the RHD irruption in 1990, the griffon vulture relied almost exclusively on carcasses of livestock and only included the rabbit anecdotally^75^. However, due to the extensive closure of large feeding stations as a result of European sanitary regulations^76^,^77^, small prey items, including rabbits, increased significantly^30^,^39^. Griffon vultures dominate Egyptian vultures and other smaller scavengers at carcasses^29^,^48^. Therefore, our model assumed that if a griffon vulture finds a rabbit carcass it consumes the resource completely, impeding access by Egyptian vultures.

Finally, the daily biomass consumption of griffon and Egyptian vultures was calculated based on the methodology described by^78^ and followed by^79^ and^80^. For both species, two estimates were calculated: 1) one for territorial individuals that breed successfully and 2) the other for both territorial individuals who failed in breeding and non-territorial individuals. In the first case, it is assumed that during the chick-rearing period the consumption increases by up to 30% due to the increase in energetic requirements^78^. In the case of the griffon vultures, we assumed that only 25% of the total population breeds (50% of the griffons in the study area are not breeders and productivity per female is 0.25 chicks/year, authors unpublished).

### Modeling future conservation policies

To determine how population viability may vary depending on different potential conservation polices and future management scenarios, we used a Box–Behnken design^81^. The Response Surface Methodology, to which the Box–Behnken design belongs, is a set of mathematical and statistical techniques used to model and analyze questions in which a variable of interest is influenced by others. The Box–Behnken designs make the estimation of the coefficients of first and second order more efficient than other designs. We specifically designed variations in two factors: food (rabbit carcasses) availability and mortality associated with daily foraging activity outside of the protected area. Food availability was measured as a percentage of the initial rabbit population existing in the study area (range: 0 to 100%). The mortality rates ranged from 2.4% to 25.5% when the vulture population remained stable and 2.5% to 100% when the vulture population doubled. The projections were simulated up to 35 years with 100 repetitions by year. Finally, we performed simulations in two different scenarios relative to the long-term trend of the griffon vulture population in the study area: stability (the population found in 2014 remains constant) and increase (the population doubles linearly in 35 years).

The model was executed using MeCoSim software (free software under license) developed by the Computation Group at the University of Sevilla (GNU GPL; htpp://www.p-lingua.org).

## Additional Information

**How to cite this article**: Cortés-Avizanda, A. *et al.* Modeling the consequences of the demise and potential recovery of a keystone-species: wild rabbits and avian scavengers in Mediterranean landscapes. *Sci. Rep.* **5**, 17033; doi: 10.1038/srep17033 (2015).

## Supplementary Material

Supplementary Information
