# Supplementary material for: Modeling the consequences of the demise and potential recovery of a keystone-species: wild rabbits and avian scavengers in Mediterranean landscapes
Source: Sci Rep. 2015 Nov 23;5:17033. doi: 10.1038/srep17033 (PMC4655486; doi:10.1038/srep17033)

Supplementary Information

Modeling the consequences of the demise and potential recovery of a keystone-species: wild rabbits and avian scavengers in Mediterranean landscapes

Ainara Cortés-Avizanda[1,2,*], Maria Àngels Colomer[3], Antoni Margalida[4,5], Olga Ceballos[2] & José Antonio Donázar[1]

[1]*Department of Conservation Biology, Estación Biológica de Doñana (CSIC), Americo Vespucio s/n, E-41092 Sevilla, Spain.*

[2]*Infraestruturas de Portugal Biodiversity Chair, CIBIO-InBio. Campus Agrário de Vairão, Rua Padre Armando Quintas 7, 4485-661 Vairão, Portugal.*

[3]*Department of Mathematics, University of Lleida, Faculty of Life Sciences and Engineering, Av. Alcalde Rovira Roure 191, 25198 Lleida, Spain.*

[4]*Department of Animal Production (Division of Wildlife), Faculty of Life Sciences and Engineering, University of Lleida, Av. Alcalde Rovira Roure 191, 25198 Lleida, Spain.*

[5]*Division of Conservation Biology. Institute of Ecology and Evolution. University of Bern. Baltzerstrasse 6, 3012 Bern, Switzerland.*

*Corresponding Ainara Cortés-Avizanda: cortesavizanda@gmail.com; acortes@cibio.up.pt

Supplementary Materials and Methods

Available biomass from wild rabbit carcasses

There are three sources of variation influencing the size of wild rabbit populations: 1) the decreased linked to RDH of 90% (1, 2,3and references therein); 2) the intra-annual (monthly) variability, which determines oscillations of around 50% in the extreme (1, 4,5); and 3) the inter-annual (yearly) variability, which only yields around 10% variation from one year to the next (6, 7). In this study we considered the greatest sources of variability, i.e. 1) and 2). In particular, we calculated the availability of rabbit biomass in two main periods: before and after RDH. In both periods, intensive field work was carried out and we obtained monthly data on the demographic parameters of wild rabbit populations in the study area (age structure, productivity and variations in population size). The first part of this study was carried out from 1983-1985, before the Rabbit Hemorrhagic Disease outbreak. A total of 732 rabbits were captured, 241 of which were active breeding females (> 1000 g weight / > 4 months old). From 1993 to 1995, we captured 1070 rabbits (244 active breeding females) (O. Ceballos unpublished data). Newborn rabbits spend their first 3-4 weeks of life underground. Therefore, we calculated the rabbit population pyramids monthly from individuals of >1 month-old (i.e. delaying the incorporation of newborns into the population by one month).

We estimated the monthly death rate from data obtained using capture/recapture methods and night transects. We compared the expected population increase in the absence of mortality (obtained from the data of age structure and productivity) with the monthly increase observed between consecutive periods. Accordingly, an average weight of 500g was assigned to each rabbit carcass from April to August. Adults

represent only 20-25% of the population and the mortality rates in juveniles and sub-adults are very high (Table S1), with the potential to exceed 90% (8-10). Moreover, since the periods of activity of the wild rabbit are basically nocturnal (11) and in the study area they remain inside warrens during the day, it has been estimated that 50% of the carcasses would remain available in the open for scavengers.

On the basis of these data, the monthly estimated biomass available before the RHD irruption (until 1990) was estimated at 172,812.5 kg calculated from April to August based on an initial population of 200,000 rabbits in March, corresponding to a density of 4 rabbits/ha. The initial wild rabbit population after 1991 was estimated to be 10% of this number (authors' unpublished data; see also references in 2) and the available biomass was estimated at 32,340 kg.

Population Dynamic P-system Model

The Population Dynamics P-system Models (PDP) (12) are a variant of P-system models and belongs to a family of computational models developed by (13). The PDP models (12) are discrete models inspired by the functioning of cells. Cells are able to run multiple processes in parallel in a perfectly synchronized manner making them good candidates for modeling complex problems. In short, we can consider the cell as a closed space separated from the medium by a membrane. The interior of the cell can be differentiated into areas where organelles are able to perform (in parallel) different interrelated and perfectly synchronized processes. Based on this simplification of a cell, the components of a P-System, and therefore a PDP model, are made up of: a number of environments (medium in which the cell is located), the structure of cell membranes (different spaces within the cell), initial alphabet (organelles) and evolution rules (processes).

According to (12) PDP and multi-agent models (15) bear much similarity; for example, they both allow the study of complex problems with different interacting agents (processes). In the case of multi-agents, it is necessary to sequence the process, while this is not necessary in PDP models. In the latter, the interacting processes can run in parallel, which is an important advantage to PDP models compared to multi-agent models.

Within each environment, the cell is composed of a series of membranes with a specific organization depending on the model. The membranes are labeled to distinguish them (by means of a subscript) and have a polarization (superscript), which can be neutral, positive or negative. While the labels are constant, polarizations vary. In the spaces formed by the membranes objects appear that evolve through rules. Objects can be found in the "cell" or in the "environment", so rules are differentiated between internal and external. A model will be defined when all four components are defined. The number of environments is often associated with the number of geographic areas in which the study area is divided to define the structure of cell membranes (in our case one).

(12) suggest starting with the simplest membrane structure, one outer membrane and one inner, which can be extended if necessary. To define the starting alphabet, an object is associated with each model input. In our case, within the space bounded by the skin membrane, there is only one object, Y_1 , that holds the information linked to the simulated year. This is used to control the quantity of food (in terms of animal biomass) that griffon vultures can obtain from food biomass provided by rabbits. One object XA_j is associated for the Egyptian vulture and the index indicates the age of the individual. qa_j is the number of existing griffons and j the age. Each vulture that exists at the initial moment, qb_1, is

associated with one object XB. Each gram of biomass from rabbits is associated with an object M. co_1, A_1, are objects that hold the month and I_1, Y_1 hold the year. G_0, GF_0 are used to account for vulture movements outside of the Natural Park, monthly and annually, respectively. R is a counter that allows us to synchronize the model. Finally, and as was detailed in Methods, the evolution rules are applied to five sequenced modules, detailed below.

Model development (according to [12])

Stage 1: Defining and clearly limiting the objective proposed and the interest of the model.

By means of the modeling procedure, we aim to disentangle how long-term population trends of a long-lived vulture are influenced by the demise of keystone species (as a consequence of RHD in wild rabbits since 1990) and an increase in the risk of non-natural mortality mainly due to the consumption of illegal poisoned baits (as a consequence of an increase in frequency of foraging excursions outside of the protected area of the Park).

Stage 2: Description of the processes to be modeled as well as the interaction between them and other processes.

The modeling procedure will be basic: mortality, reproduction and competition with griffon vultures (for food resources) (Fig. S1). The species can forage outside of the study area, but their presence will increase outside the Park when food availability is insufficient to meet the energetic requirements of the focal species. Thus, for each individual the model will take into account the times that the individual foraged outside of the Park, affecting their fitness (i.e. breeding output, Table S2) and the risk of non-natural mortality (Table S3).

Stage 3: The input of the model and the parameters involved.

Model inputs can be grouped into four types:

Size of the population. The starting point is the Egyptian vulture population in the Natural Park in 1980. The Eurasian griffon vulture is considered the species that competes food. The population dynamics of this species have not been modeled but their annual population has been incorporated as an input in the model. The griffon population foraging in the protected area was calculated from the density of prospecting birds observed in 2005 by (16). Long-term changes in this value (Fig. S2) were estimated on the basis of the changes in the population breeding in the upper Ebro Valley (northern Spain) from 1979 to 2013 (authors and C. Fernández and P. Azkona, unpublished).

Food. The principal trophic resource for the Egyptian vulture population in the study area is the carcasses of the wild rabbits in the Natural Park. The rabbit population trend was obtained through censuses (see above) and the biomass available was also estimated (see above). The distribution of the food over six months (Table S1) is not uniform. The Egyptian vultures breed in the Natural Park such that an input will be the monthly distribution of biomass. Trophic requirements for scavengers vary depending on the time of year and whether or not the animal is breeding. Thus, we need to know the average percentage of females that reproduce annually and the consumption will depend on the month and breeding status of the individual (Table S4).Wild rabbit makes up part of the vulture diet and its proportion in the diet varies over time (see above and Table S4).

Parameters depending on how frequently the vulture leaves the park. Some of the Egyptian vultures that breed in the park area can visit neighboring areas. This implies a cost for the individuals resulting in an increased mortality risk due to the above-mentioned human pressures and, in addition, an energy expense that leads to a decrease in breeding success. Both breeding success and mortality (Tables S2 and S3) vary with the time at which the individual leaves the park, with the range of estimated values being 0.5-0.8 for breeding success (low breeding success when the number of visits outside the park is about >151

days/year) and increasing mortality to 0.2% (0.2% when an individual visits outside areas 21-30 times per month).

General parameters. In this last section, we describe the general biological parameters of the species such as life expectancy (25 years), sex ratio (0.5), age at first breeding attempt (8 years), natural mortality of pre-adults (0.1) and adults (0.05), maximum load of the species in the park (50 pairs), probability of leaving the park when sufficient feeding resources exist inside the Natural Park (0.2) and the percentage of food obtained outside the protected area (0.2%) (see Table S5).

Stage 4: Designing a model scheme that describes the sequencing and parallelization of the processes.

The first step is to attempt sequence processes. The PDP models can operate in parallel so it is possible to model more than one process at a time. Sequencing, if possible, allows for better organization and therefore, facilitates the control model. It begins executing the process of natural mortality and additional non-natural mortality linked to foraging movements that individuals perform in highly humanized areas outside the park (module 1). In the second module, the feeding process runs depending on the availability of food in the Natural Park. According to this module, Egyptian vultures may or may not be obligated to abandon the park as a function of food availability. In the next module, excursions outside of the park due to a lack of food resources are counted. The feeding process and recorded exits from the park are made daily each month, and thus this is repeated 30 times (modules 2 and 3). In the end, we obtain the number of times each bird leaves the park in search of food. This is the input for module 4, which addresses mortality due to the ingestion of food with poison. At the end of the month, the model runs module 1 again and this loop is repeated six times, once per month. At the end of the sixth month the number of times the animal has exited the park in the search of food during the year is recorded. This parameter

is the input for module 5 for the reproductive process. The completion of the module 5 ends the execution of one year. The loop should be repeated as many times as necessary to simulate years.

Stage 5: Designing the model.

The model will be complete when each of the components is defined: the number of environments, the membrane structure, the initial alphabet and the evolution rules. There is only one geographic area and thus only one environment is defined.

According to (12), the model begins with the simpler membrane structure, $\boldsymbol{\mu} = [[\quad]_{\mathbf{1}}]_{\mathbf{0}}$, composed of a skin and a membrane. The model has been fully developed without increasing the number of membranes.

The initial alphabet is formed by the objects associated with the input of the model,

$$\boldsymbol{\mu_0} = \{\boldsymbol{Y_1}\}$$

$$\boldsymbol{\mu_1} = \left\{\boldsymbol{XA_j^{qa_j}}, \mathbf{1} \leq \boldsymbol{j} \leq \boldsymbol{g_2}, \boldsymbol{XB^{qb_1}}\right\} \cup \left\{\boldsymbol{M^{Bio(1,1)}}\right\} \cup \{\boldsymbol{co_1}, \boldsymbol{I_1}, \boldsymbol{R_0}\} \cup \left\{\boldsymbol{A_1}^{\mathbf{3}\boldsymbol{N_1}}, \boldsymbol{Y_1}^{\mathbf{30}\boldsymbol{qb_1}}\right\} \cup \left\{\boldsymbol{G_0}^{\boldsymbol{qa_j}}, \boldsymbol{GF_0}^{\boldsymbol{qa_j}}, \mathbf{1} \leq \boldsymbol{j} \leq \boldsymbol{g_2}, \boldsymbol{XB^{qb_1}}\right\}$$

Most of the objects are found in the inner membrane labeled with 1. Each Egyptian vulture is associated with an object of type $\boldsymbol{XA_j}$ whose index indicates the age. Similarly way each griffon vulture be joined $\boldsymbol{XB}$ object. In this case, no index appears, given in this case we do not take into account the age of the individual. The amount of food is measured in grams, such that each gram of rabbit available is represented by an object type $\boldsymbol{M}$.

In addition to the objects that are associated with the input model, other objects are needed The object $\boldsymbol{I_j}$ allows the control of the simulation year and processes associated with the

year being simulated, $\boldsymbol{Y_j}$. The month is controlled with the object $\boldsymbol{co_i}$. The objects $\boldsymbol{G_i}$ and $\boldsymbol{GF_i}$ hold the number of movements made for each animal outside of the park every month and every year, respectively. Finally, object $\boldsymbol{R_i}$ acts as a counter and allows the synchronization of the model.

Evolution rules

The parameters of the model are defined in Table S5.

Module 1. Natural and non-natural mortality

Some of the breeding Egyptian vultures will die of natural causes (m) or from the consumption of poisoned baits ($po \cdot poison$). Based on the probabilities of these events, some objects of type $\boldsymbol{XA}$ will dissolve and other objects associated with animals that survive evolve to $\boldsymbol{YA}$ or $\boldsymbol{YAo}$ objects depending on whether they leave the park or not, respectively.

.

$$\boldsymbol{r_1 \equiv \left[XA_j, G_0 \xrightarrow{\frac{m+po\cdot poison}{6}} GM \right]_1^0 , \quad g_1 \le j \le g_2.}$$

$$\boldsymbol{r_2 \equiv \left[XA_j, G_0 \xrightarrow{\left(1-\frac{m+po\cdot poison}{6}\right)\cdot\frac{(1-po)}{1-po\cdot poison}} YA_j, G_0 \right]_1^0 , \quad g_1 \le j \le g_2.}$$

$$\boldsymbol{r_3 \equiv \left[XA_j, G_0 \xrightarrow{\left(1-\frac{m+po\cdot poison}{6}\right)\cdot\frac{(po-po\cdot poison)}{1-po\cdot poison}} YAo_j, G_0 \right]_1^0 , \quad g_1 \le j \le g_2.}$$

For immature Egyptian vultures, only natural death is considered ($\boldsymbol{mf}$), and as in the case of adults, some of the objects associated disappear and the rest of the objects evolve.

$$\boldsymbol{r_4 \equiv \left[XA_j, G_0 \xrightarrow{\frac{mf}{6}} GM \right]_1^0, \qquad 1 \leq j < g_1.}$$

$$\boldsymbol{r_5 \equiv \left[XA_j, G_0 \xrightarrow{1-\frac{mf}{6}} YA_j, G_0 \right]_1^0, \qquad 1 \leq j < g_1.}$$

The existing number of vultures in the park each year is an input in the model, and therefore mortality is not considered in this case. The objects associated with the vultures only evolve.

$$\boldsymbol{r_6 \equiv [XB \longrightarrow YB]_1^0, \qquad 1 \leq j < g_1.}$$

$\boldsymbol{R_i}$ is a counter that allows the steps of the model to be registered and thus synchronized.

$$\boldsymbol{r_7 \equiv [R_0 \longrightarrow R_1]_1^0.}$$

Module 2. Feeding

The feeding process occurs daily; therefore the rules of this module are repeated 30 times.

The amount of food consumed by each vulture, $\boldsymbol{fa}$, depends on the time of year (month, index $\boldsymbol{k}$), and the object $\boldsymbol{A_k}$ in its index stores the information of the month. When the object associated with an Egyptian vulture can access the necessary food, it evolves to object $\boldsymbol{ZA}$

$$\boldsymbol{r_8 \equiv \left[A_k, YA_j, M^{\frac{fa_k}{30}\cdot(1-food)}\right]_1^0 \longrightarrow \left[ZA_j, A_k\right]_1^+, 1 \le j \le g_2, 1 \le k \le 6.}$$

$\boldsymbol{(1-food)}$ is the proportion of food consumed by each animal in the park

Birds that have left the park do so because they obtained little resources within the park. Thus, they need to forage outside of the park to obtain the necessary resources to meet their energetic requirements.

$$\boldsymbol{r_9 \equiv \left[A_k, YAo_j, M^{\frac{fa_k}{30}\cdot(1-food)\cdot(1-fo)}\right]_1^0 \longrightarrow \left[ZA_j, A_k\right]_1^+, 1 \le j \le g_2, 1 \le k \le 6.}$$

Similarly, there is a feeding rule for griffon vultures. In this case, the amount of food depends on the month but not the year of simulation. The information is stored in the index object $\mathbf{Y}$.

$$\boldsymbol{r_{10} \equiv \left[YB, Y_i, M^{\frac{fb_k}{30}\cdot(Weight_i)}\right]_1^0 \longrightarrow [ZB]_1^+, 1 \le k \le 6, 1 \le i \le Year.}$$

$\boldsymbol{Weight_i}$is the propotion of rabbits consumed by griffons during the month $\boldsymbol{i}$. The amount of food consumed by each griffon, $\boldsymbol{fb_k}$**,** depends on the time of year

To control the model we have the $\boldsymbol{R}$ object, which counts steps.

$$r_{11} \equiv [R_{4\cdot i-3}]_1^0 \longrightarrow [R_{4\cdot i-2}]_1^+, 1 \le i \le 30.$$

Module 3. Accounting for exists from the park

The objects associated with vultures with insufficient food has not evolved, and the rest evolve to ZA' type objects and generate a new object, G', to account for the times that the animal left the park.

$$r_{12} \equiv \left[YA_j \longrightarrow ZA'_j, G'\right]_1^+, 1 \leq j \leq g_2.$$

$$r_{13} \equiv \left[YAo_j \longrightarrow ZA'_j, G'\right]_1^+, 1 \leq j \leq g_2.$$

The objects associated with vultures that have not eaten in the park evolve but do not create new objects.

$$r_{14} \equiv [YB \longrightarrow ZB']_1^+.$$

Objects that had evolved in the previous step (module 2) evolve but do not create new objects.

$$r_{15} \equiv \left[ZA_j \longrightarrow ZA'_j\right]_1^+, 1 \leq j \leq g_2.$$

$$r_{16} \equiv [ZB \longrightarrow ZB']_1^+.$$

The counter continues to record steps.

.

$$r_{17} \equiv [R_{4\cdot i-2} \rightarrow R_{4\cdot i-1}]_1^+, 1 \leq i \leq 30.$$

To ensure the consistency of the model, in this step only changes membrane charge and the objects do not evolve,

$$r_{18} \equiv [R_{4\cdot i-1}]_1^+ \rightarrow [R_{4\cdot i}]_1^0, 1 \leq i \leq 29.$$

When the membrane labeled with 1 changes to a negative polarization, this indicates the month is complete.

$$r_{19} \equiv [R_{119}]_1^+ \longrightarrow [\#]_1^-.$$

To finish the process of feeding, the ZA' objects evolve to YA objects ready to begin the process of feeding the next day.

$$r_{20} \equiv \left[ZA'_j \longrightarrow YA_j\right]_1^0, 1 \ \leq j \leq g_2.$$

$$r_{21} \equiv [ZB' \longrightarrow YB]_1^0.$$

The G and GF objects record the times that the animals leave the park monthly and annually. If an object generates a G' , this indicates that there was a new exit and so a unit should be added to each object.

$$r_{22} \equiv \left[G_j, G', GF_i \longrightarrow G_{j+1}, GF_{i+1}\right]_1^0, 0 \leq j \leq 29, 0 \leq i \leq 179.$$

$$r_{23} \equiv [R_{4\cdot i} \longrightarrow R_{4\cdot i+1}]_1^0, 1 \leq i \leq 29.$$

At the end of the month, some of the objects associated with animals evolve and the outputs of the animals in the park are counted, the co object index should increase by one to indicate that the following month has begun. When the sixth month is reached this object index is reset to 1 and the index of the object I which records the year increases by one.

$$r_{24} \equiv \left[ZA'_j \longrightarrow YA_j\right]_1^-, 1 \ \leq j \leq g_2.$$

$$r_{25} \equiv [ZB' \longrightarrow \#]_1^-.$$

$$r_{26} \equiv \left[G_j, G', GF_i \longrightarrow G_{j+1}, GF_{i+1}\right]_1^-, 0 \leq j \leq 29, 0 \leq i \leq 179.$$

$$r_{27} \equiv \left[I_j, co_i\right]_1^- \longrightarrow I_j, co_{i+1}[\quad]_1^-, 1 \leq j \leq Year, 1 \leq i \leq 5.$$

$$r_{28} \equiv \left[I_j, co_6\right]_1^- \longrightarrow I_{j+1}, co_1[\quad]_1^-, 1 \leq j \leq Year.$$

The objects associated with the food that remained were dissolved.

$$r_{29} \equiv [M \longrightarrow \#]_1^-.$$

$$r_{30} \equiv [A_i \longrightarrow AA_i]_1^-, 1 \le i \le 6.$$

Module 4. Mortality from consumption of poisoned food when food is insufficient in the park

The mortality index (mo_i) varies depending on the number of times, (Lmo_i) that the animal leaves the park. When an animal dies, the associated object dissolves and is recorded in the GM object. G_m resets to G_0 if the object does not dissolve.

$$r_{31} \equiv \left[YA_j, G_m\right]_1^- \xrightarrow{mo_i} [GM]_1^+, g_1 \le j \le g_2, Lmo_i \le m < Lmo_{i+1}, 1 \le i \le 5.$$

$$r_{32} \equiv \left[YA_j, G_m\right]_1^- \xrightarrow{1-mo_i} \left[YA'_j, G_0\right]_1^+, g_1 \le j \le g_2, Lmo_i \le m < Lmo_{i+1}, 1 \le i \le 5.$$

$$r_{33} \equiv \left[YA_j, G_0\right]_1^- \longrightarrow \left[YA'_j, G_0\right]_1^+, g_1 \le j \le g_2.$$

$$r_{34} \equiv \left[YA_j, G_m\right]_1^- \longrightarrow \left[YA'_j, G_0\right]_1^+, 1 \le j < g_1, 0 \le m \le 30.$$

Module 5. Reproduction and restoration of the initial configuration

This is the last step that takes place at the end of the month. This will differentiate the first five months (of the six) and the last of the years in which the reproductive process is added.

When objects associated with the year and month of simulation enter the inner membrane objects that are associated with the Egyptian vulture population, biomass, month, and year are generated and the counter is reset. In the case of the last month, objects a are also generated to allow for the monitoring of the maximum load.

$$r_{35} \equiv I_j, co_i[\quad]_1^+ \to \left[XB^{qb_j}, Y_j^{30\cdot qb_j}, co_i, I_j, A_i^{10\cdot N_1}, M^{Bio_{j,i}}, R_0\right]_1^+, 2 \le i \le 6, 1 \le j \le Year.$$

$$r_{36} \equiv I_j, co_1[\quad]_1^+ \longrightarrow \left[XB^{qb_j}, Y_j^{30\cdot qb_j}, co_1, I_j, A_i^{10\cdot N_1}, M^{Bio_{j,1}}, R_0, a^d\right]_1^+,\ 1 \le j \le Year.$$

When the last year of simulation is reached, it is only necessary to create objects that will be used to control the maximum load of Egyptian vultures.

$$r_{37} \equiv I_{Year+1}, co_1[\quad]_1^+ \longrightarrow [a^d]_1^+ .$$

While not reaching the last month, the objects YA' evolve to XA; when the end of the annual cycle is reached the object YA' evolves to XA' to begin the process of reproduction.

$$r_{38} \equiv \left[YA'_j, AA_i \longrightarrow XA_j\right]_1^+, 1 \le j \le g_2, 1 \le i \le 5 .$$

$$r_{39} \equiv \left[YA'_j, AA_6 \longrightarrow XA'_j\right]_1^+, 1 \le j \le g_2 .$$

$$r_{40} \equiv \left[GF_m, GM \longrightarrow \#_j\right]_1^+, 0 \le m \le 180 .$$

Animals that reproduce successfully evolve and create new objects corresponding to descendants. Breeding success depends on the number of excursions that the animal has made outside of the park.

$$r_{41} \equiv \left[XA'_j, GF_m, a\right]_1^+ \xrightarrow{g_3\cdot r_m} \left[XA_{j+1}, XA_1, GF_0^2, GF'_m, G_0\right], g_1 \le j < g_2, 0 \le m \le 180 .$$

$$r_{42} \equiv \left[XA'_j, GF_m, a\right]_1^+ \xrightarrow{1-g_3\cdot r_m} \left[XA_{j+1}, GF_0^2, GF'_m\right], g_1 \le j < g_2, 0 \le m \le 180 .$$

$$r_{43} \equiv \left[XA'_j, GF_m\right]_1^+ \rightarrow \left[XA_{j+1}, GF_0\ , GF'_m\right], 1 \le j < g_1 - 1, 0 \le m \le 180 .$$

$$r_{44} \equiv \left[XA'_{g_1-1}, GF_m, a\right]_1^+ \rightarrow \left[XA_{g_1}, GF_0\ , GF'_m\right], 0 \le m \le 180 .$$

$$r_{45} \equiv \left[XA'_{g_2}, G_0, GF_m, a\right]_1^+ \xrightarrow{g_3\cdot r_m} \left[XA_1, GF_0\ , GF'_m, G_0\right], 0 \le m \le 180 .$$

$$r_{46} \equiv \left[XA'_{g_2}, G_0, GF_m, a\right]_1^+ \xrightarrow{1-g_3\cdot r_m} [\#], 0 \le m \le 180 .$$

The object GF'_m is a copy of the time that the Egyptian vultures leave the park, which is only used to record the output of the model.

Finally, the remaining objects are removed and the polarization of the inner membrane is changed.

$$r_{47} \equiv [R_0]_1^+ \to [R_0].$$

$$r_{48} \equiv [AA_i \to \#]_{1,}^0, 1 \leq i \leq 6.$$

$$r_{49} \equiv \left[XA'_j, G_0, GF_m \to \#\right]_{1,}^0, g_1 \leq j \leq g_2, 0 \leq m \leq 180.$$

$$r_{50} \equiv \left[a \to \#\right]_{1,}^0.$$

$$r_{51} \equiv \left[GF'_m \to \#\right]_{1,}^0, 0 \leq m \leq 180.$$

Stage 6: Graphical representation of the configurations that represent the execution of a cycle of the model.

Based on the initial configuration and following the rules and steps discussed in the previous section, the different settings that appear in the model run are shown (Fig. S3). Running a year involves 744 steps, and 51 types of rules representing 24849 unfolded rules.

Stage 7: Designing the simulator.

A computer simulator is required to facilitate the implementation of the model and to study the population dynamics for a range of hypothetical scenarios. MeCoSim was used to design the simulator. MeCoSim, designed by the Natural Computing research group at Seville University, is freely available for download (14, 17). The values of the input parameters can be modified directly from the screen, making it easier to study the behavior of the focal species in a given scenario. The model output for each simulated year provides

the average number and deviation of young and adult birds and the average number of visits outside the park by individual and year.

References

1. Villafuerte, R., Calvete, C., Gortázar, C. & Moreno, S. First epizootic of rabbit hemorrhagic disease in freeliving populations of (*Oryctolagus cuniculus*) at Doñana National Park, Spain. J. Wildl. Dis. **30**,176-179 (1994).

2. Villafuerte, R., Calvete, C. Blanco, J. C. & Lucientes, J. Incidence of viral hemorrhagic disease in wild rabbit populations in Spain. *Mammalia* **59**, 651–659 (1995).

3. Delibes-Mateos, M., Ferreira, C., Carro, F., Escudero, M. A. & Gortázar, C. Ecosystem effects of variant Rabbit Hemorrhagic Disease Virus, Iberian Peninsula. *Emerging Infectious Diseases* **20**, 12. (2014).

4. Soriguer, R. Biología y dinamica de una poblacion de Conejos (*Oryctolagus cuniculus*) en Andalucia Occidental. Acta Vertebrata. Resvista de Vertebrados de la Estación Biológica de Doñana (CSIC). Sevilla. Spain. (1981)

5. Villafuerte, R. Riesgo de predación y estrategias defensivas del conejo (*Oryctolagus cuniculus*) en el Parque Nacional de Doñana. Tesis Doctoral. Univ. Córdoba. Spain. (1994)

6. Beltrán, J.F. Temporal abundance pattern of the wild rabbit in Doñana, SW Spain. *Mamalia* **55**, 591-599. (1991)

7. Moreno, S. et al. Long-term decline of the European wild rabbit (*Oryctolagus cuniculus*) in south-western Spain. CSIRO *Wildl. Res.* **34**,652-658 34 (2007)

8. Calvete, C. Estrada, R. Villafuerte, R. Osácar, J. J.& Lucientes, J. Epidemiology of viral haemorrhagic disease and myxomatosis in a free-living population of wild rabbits. *Vet. Rec.* **150**, 776–782 (2002).

9. Tyndale-Biscoe, C.H. & R.M. Williams. A study of natural mortality in a wild population of the rabbit, *Oryctolagus cuniculus*. *New Zeal. J. Dairy Sci.* **36**, 561-580 (1955).

10. Wheeler, S. H. & King D. R.The European rabbit in Southwestern Australia. III. Survival. *Aust. Wildlife Res.* **12**, 213-225 (1985).

11. Kolb, H.H. Circadian activity in the wild Rabbit (*Oryctolagus cuniculus*). *Mammal Rev.* **16**,145-150 (1986).

12. Colomer, M.A., Margalida, A. & Pérez-Jiménez, M. Population dynamic P system (PDP) models: a standardized protocol for describing and applying novel bio-inspired computing tools. *PLoS ONE* **8**, e60698 (2013).

13. Păun, G. Computing with membranes. *J Comput Syst Sci* **61**, 108–143 (1998).

14. Díaz, D., Pérez-Hurtado, I., Pérez-Jiménez, M. J. & Riscos, A. A P-lingua programming environment for Membrane Computing. *LNCS* **5391**, 187–203. (2009).

15. Grimm, V. & Railsback, S.F. Individual-based modeling and ecology. Princeton University Press, Princeton (2005)

16. Cortés-Avizanda, A., Jovani, R., Donázar, J.A. & Grimm, V. Birds sky networks: How do avian scavengers search for carrion resource. *Ecology* **95**, 1799-1808 (2014).

17. Pérez-Hurtado, I., Valencia, L., Pérez-Jiménez, M. J., Colomer, M. A. & Riscos, A. MeCoSim: A general purpose software tool for simulating biological phenomena by means of P Systems. in: *Proceedings 2010 IEEE Fifth International Conference on Bio-inspired*

Computing: Theories and Applications (eds Li, K., Tang, Z., Li, R., Nagar, A.K. & Thamburaj, R.) 637–643. Vol 1. (Changsha: IEEE Press. 2010).

18.

Table S1. Monthly distribution (%) of the biomass available from rabbit carcasses in the Natural Park.

**Period**	**March**	**April**	**May**	**June**	**July**	**August**
Before 1990	27.13	27.03	18.41	15.23	9.12	3.08
After 1990	21.52	11.05	29.38	26.78	7.93	3.34

Table S2. Variation in the breeding success of Egyptian vultures in relation to the yearly number of foraging trips outside the Natural Park.

Movements outside	Breeding success
0	0.8
1-30	0.7
31-60	0.65
61-90	0.6
91-120	0.55
121-150	0.5
151-180	0.5

Table S3. Variation in the increase in the mortality rate of Egyptian vultures according to the number of monthly foraging trips outside the Natural Park.

Movements	Increase in the probability of mortality by poisoning
0	0
1-5	0.15
6-10	0.15
11-20	0.2
21-30	0.2

Table S4. Individual monthly energetic requirements (gr) of Egyptian vultures and griffon vultures according to their breeding status (breeding vs not breeding).

	Breeding	%Females	March	April	May	June	July	August
Egyptian vulture	Yes	80	6200	7650	8450	9264	7840	6300
Egyptian vulture	No	20	6200	6200	6200	6200	6200	6200
Eurasian griffon vulture	Yes	25	21700	26000	28750	31500	26600	21700
Eurasian griffon vulture	No	75	21700	22775	24000	25300	24725	21700

Table S5. Definitions of the Egyptian vulture demographic parameters included in the model.

	**Demographic parameters**
g_1	Age at first breeding (years)
g_2	Average life expectancy (years)
g_3	Sex ratio
m	Natural adult mortality (per one)
mf	Natural preadult mortality (per one)
d	Maximum number of adults (individuals)
	**Foraging movements**
po	Probability of leaving the Park when resources exist (per one)
poison	Mortality increase as a consequence of ingestion of contaminated food outside the Natural Park (per one)
fo	Percentage of total food taking out (with respect to available resources)
$food$	Percentage of the energetic requirements obtained outside the Park
$r_i, 1 \leq i \leq 7$	Breeding success according to the times (i) that an individual forages outside the Park
$mo_i, 1 \leq i \leq 5.$	Mortality increase related to the ingestion of poison baits according to the times (i) an individual forages outside the Park (per one)
$Lmo_i, 1 \leq i \leq 5$	Ends of the intervals defining groups performed with variable number of movements outside the Park
	**Disease**
disease	1 when the rabbits are affected by a disease, 0 when they are not affected.
	**Feeding**
$Bioma$	Weight (gr) of available rabbits as food
$fa_i, 1 \leq i \leq 6$	Egyptian vulture's monthly (i) energetic requirements (gr)

$fb_i, 1 \leq i \leq 6$	Eurasian griffon vulture's monthly (i) energetic requirements (gr)
$weight_i$	Percentage of rabbits in the Eurasian griffon vulture diet in month i

Figure S1. Scheme of the biological process involved in the model.

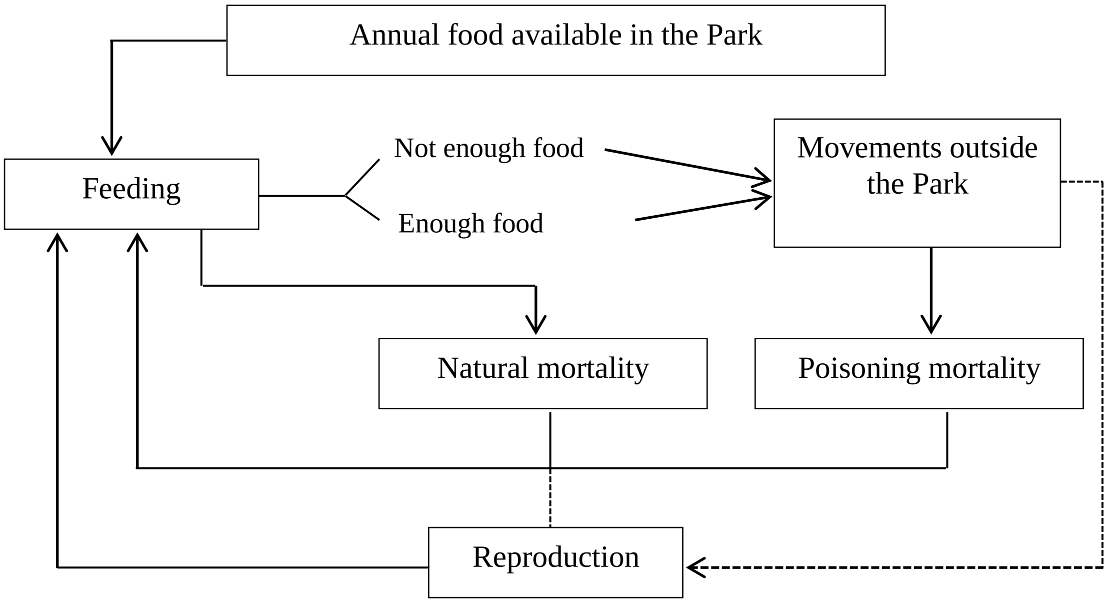

Figure. S2. Estimation of the long-term variation in the number of Eurasian griffon vultures prospecting the Natural Park.

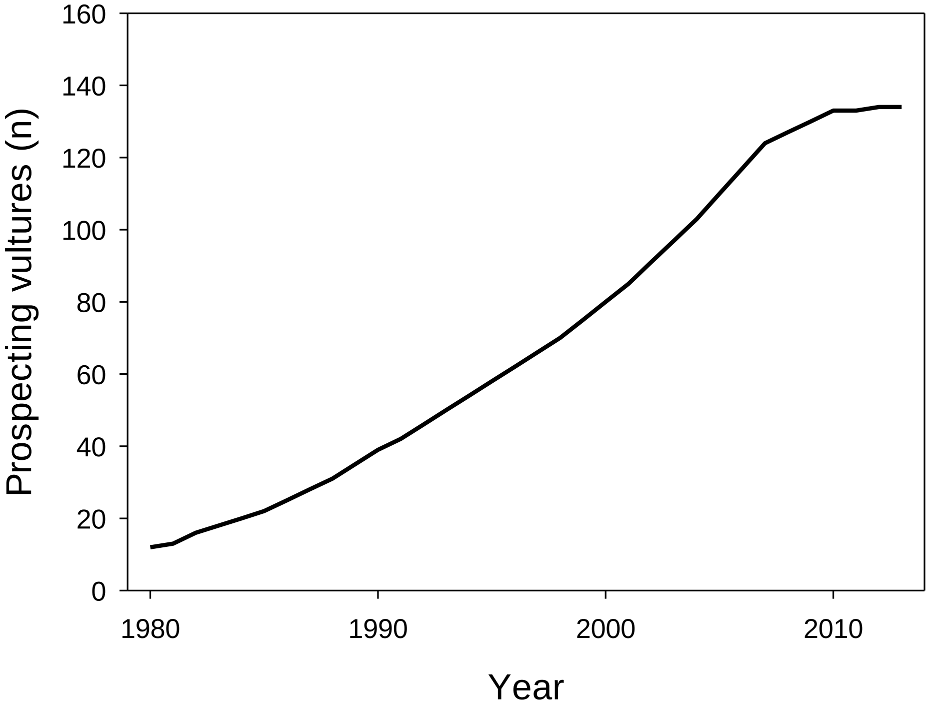

Figure S3. Configurations generated by the model through the application of the evolution rules.

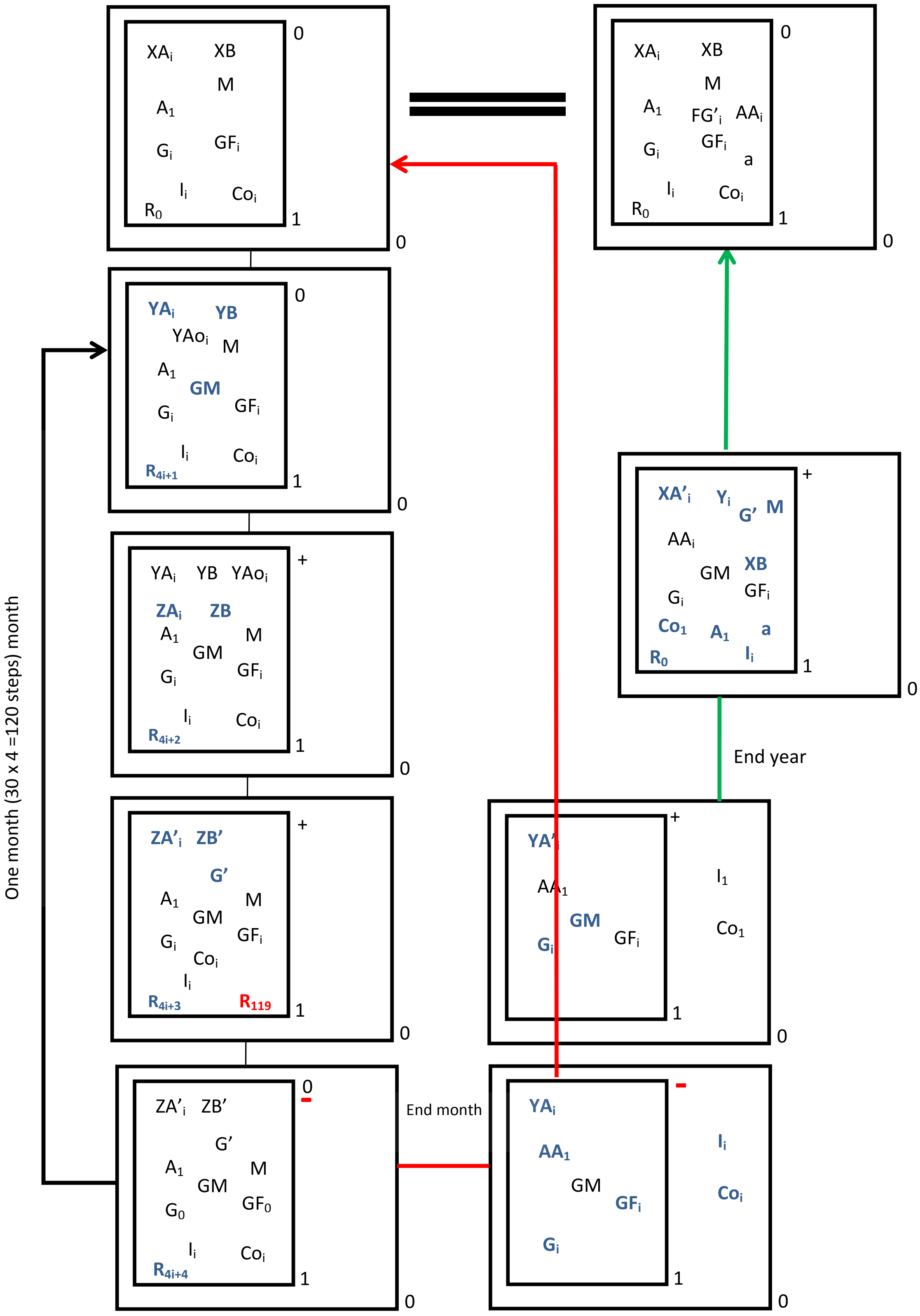

Supplement: Supplementary Information [file srep17033-s1.pdf]
